# Corticosteroids, Plasmapheresis, Argatroban, Rituximab, and Sirolimus Provided Clinical Benefit for Catastrophic Antiphospholipid Syndrome in a Patient with a History of Heparin-Induced Thrombocytopenia

**DOI:** 10.1155/2023/3226278

**Published:** 2023-02-09

**Authors:** Haytham Hasan, Ivana Surjancev, Jon A. Arnason, Shivani Garg, William Nicholas Rose

**Affiliations:** ^1^Department of Pathology, University of Wisconsin Hospital, 600 Highland Ave, Madison, WI 53792, USA; ^2^Department of Medicine, University of Wisconsin Hospital, 600 Highland Ave, Madison, WI 53792, USA

## Abstract

We report a patient with catastrophic antiphospholipid syndrome who had significant improvement after corticosteroids, plasmapheresis, argatroban, rituximab, and sirolimus. Argatroban was used instead of heparin due to a history of heparin-induced thrombocytopenia.

## 1. Introduction

Antiphospholipid syndrome (APS) is a systemic autoimmune disorder characterized by clinical manifestations including arterial and/or venous thrombosis, recurrent fetal loss, and elevated titers of antiphospholipid antibodies [1, 2]. The main target of these antibodies is the phospholipid membrane of platelets, and binding leads to platelet activation [3]. While many nuances exist, the basic diagnostic criteria include one or more episodes of venous and/or arterial thrombosis and/or obstetric complications in a patient with laboratory evidence of persistent antiphospholipid antibodies such as lupus anticoagulant, anticardiolipin, and/or anti-beta 2 glycoprotein [1, 4–8]. The subcategory of catastrophic antiphospholipid syndrome (CAPS) is defined as the acute onset of multiple thromboses in at least 3 organ systems over a period of less than one week in a patient with antiphospholipid antibodies [4].

Given its rarity and the lack of data, the optimal treatment for CAPS has not been established. The number of patients reported who received plasmapheresis for CAPS is about 100–300 [4]. Moreover, there are no randomized, prospective, or controlled clinical trials that have studied plasmapheresis (or TPE, therapeutic plasma exchange) for CAPS [4]. In addition, only a few dozen total patients who have received rituximab for either CAPS or APS have been reported [9–11]. Thus, the evidence is relatively sparse overall and especially for patients who have received both plasmapheresis and rituximab. In that context, we share our experience in order to contribute to the growing body of data regarding treatment strategies for CAPS.

## 2. Case Presentation

Our patient is a 43-year-old male who was admitted for lower extremity pain, a retiform purpuric rash on his right leg, and chest pain. He had a past history of diabetes, left leg deep vein thrombosis for which he was on fondaparinux (10 mg/day), heparin-induced thrombocytopenia (HIT), and peripheral arterial disease with a fem-fem bypass and iliac stenting for which he was on chronic baby aspirin (completed 1 year prior to presentation). HIT was diagnosed 8 months prior to presentation given platelet drop from 173 to 90 within 3 days of initiation of heparin drip and a platelet factor 4 IgG antibody was elevated to 1.572 OD. Troponin on admission was found to be mildly elevated. As part of the workup for his right leg pain and rash, a skin biopsy showed vasculopathy with microthrombi. Blood test results were also notable for a partial thromboplastin time of 83 seconds and positive beta 2 glycoprotein (IgG > 150 SGU, IgM 40 SMU) and cardiolipin antibodies (IgA > 150 SAU, IgM 69 MPL) which remained positive twelve weeks later (34 SGU, 11 SMU, >150 SAU, and 23 MPL, respectively). Lupus anticoagulant was not checked. During this hospitalization, he was treated for antiphospholipid syndrome (APS) with corticosteroids (given refractory response to chronic aspirin) and with a dose of rituximab. He was discharged with orders for 3 more doses of rituximab.

About 2 weeks after the above initial presentation, he was admitted again for a new fever of 102 F and diffuse arthralgias. On the day of admission, he developed sudden-onset symmetrical sharp pain in his shoulder joints that spread to his hips, knees, knuckles, and back. The patient also reported headache.

His blood tests were notable for severe hyperglycemia. He was treated with insulin due to concern for diabetic ketoacidosis secondary to prescribed steroids. He was also given broad-spectrum antibiotics (cefepime and vancomycin). His home prednisone was held due to concern for infection based on his diffuse arthralgias, elevated temperature, and immunocompromised status (although, in hindsight, the home prednisone should have been continued given that the actual etiology of his illness was autoimmune not infectious). Rheumatology was consulted on readmission and noted recurrence of a tender violaceous rash along his right lower extremity similar to his prior retiform rash.

A couple of days later during this same admission, the patient reported improvements in his presenting symptoms but started complaining of worsening shortness of breath. He quickly decompensated with acute hypoxic respiratory therapy, was transferred to intensive care, and required intubation and mechanical ventilation. Computed tomography (CT) showed diffuse bilateral ground glass opacities of the lungs and broncho-alveolar lavage (BAL) showed diffuse alveolar hemorrhage. Additionally, the patient's creatinine had doubled within this time period consistent with an acute kidney injury concerning for renal involvement with proteinuria on subsequent urinalysis. A faint rash was appreciated.

Given the involvement of 3 simultaneous organ systems (namely, the skin, kidneys, and lungs) along with histopathologic evidence of microthrombi in the skin biopsy and the positive serological tests that were 12 weeks apart, a diagnosis of definite catastrophic antiphospholipid syndrome (CAPS) was made as per the classification criteria [6]. Additionally, the patient had chest pain despite a negative troponin and reassuring EKG during this time though there was concern for potential progression to cardiac involvement given previously elevated troponin on prior admission and possible contribution of APS to this manifestation.

He received high-dose IV steroids for 3 days plus plasmapheresis four times with plasma as the replacement fluid (once daily on 3 consecutive days and a fourth time 3 days after the third plasmapheresis). The plasmapheresis was centrifugal, and one plasma volume was replaced per procedure. There was originally a fifth plasmapheresis procedure planned, but the patient had a severe allergic reaction during the fourth procedure; consequently, the fifth procedure was cancelled.

After the fourth plasmapheresis, the patient's respiratory status had improved, and he was extubated and returned to general medicine care with continued oxygen requirement of 6 L via nasal cannula. Shortly thereafter, he no longer required supplemental oxygen. The patient was also started on argatroban anticoagulation prophylaxis due to his past history of heparin-induced thrombocytopenia, and this anticoagulant was preferred over fondaparinux given ease of reversibility if needed. Platelet count at time of argatroban was 118 and recovered to 192 within a couple days. He had no known heparin exposures during the hospitalizations above for CAPS, and his heparin-induced thrombocytopenia scores were low risk during these hospitalizations. Additionally, hematology had been consulted on all admissions and had documented that the patient had been therapeutic and compliant with warfarin to date, with routine INR ranging 2-3 (upper limit) appropriately.

About one day after the fourth plasmapheresis, the patient was discharged from the hospital. A couple of weeks later, the patient presented with lower extremity pain and arthralgias, a severely painful rash on his left foot, dusky discoloration, and pulses that were difficult to palpate. Lower extremity swelling was also noted. Computed tomography angiography (CTA) showed a subacute superficial femoral artery occlusion. He was diagnosed with avascular necrosis (AVN) of the right tibia and right foot, likely multifactorial from his recurrent high dose steroids in setting of difficult to control APS and possibly from recurrent thrombi from antiphospholipid syndrome itself. He received 500 mg daily of pulsed IV methylprednisolone for 3 days, and his fondaparinux was increased from 10 mg to 12.5 mg daily (as hematology felt this provided more effective anticoagulation given his history when compared to warfarin).

By then, the patient had received a total of 3 doses of rituximab. He did not receive his 4^th^ dose of rituximab because cumulatively he had received 900 mg (approximately 375 mg/m^2^) and it was thought that additional rituximab would be unlikely to result in further benefit. Sirolimus was started per hematology's recommendations at a dose of 2 mg daily as he had not appropriately responded to rituximab and ongoing plasmapheresis was unsafe due to his severe allergic reaction. Additionally, there is evidence that sirolimus can be used for certain patient populations and may be protective in those with nephropathy related to APS [10, 12]. He worked with pain management and had nerve blocks to help control his pain and was ultimately discharged on an oral prednisone taper, decreasing by 10 mg every 3 days in the event that the AVN may be related to steroid exposure. Since he presented with diffuse alveolar hemorrhage and concern for CAPS despite being on mycophenolate and hydroxychloroquine, rituximab and steroids were started. He received 1 g of methylprednisolone for 3 days that was tapered to 60 mg of prednisone that was slowly tapered further.

A couple of months later, the patient developed new numbness in his hands and legs. Initially, there was concern that this could be a side effect of sirolimus; however, the numbness persisted even after this medication was stopped. The numbness was attributed to the patient's diabetes, and sirolimus was restarted.

After receiving about 6 months of sirolimus treatment, the patient went off this medication and reported that he was doing well overall and was able to exercise without worsening leg pain or worsening chest pain. His numbness persisted but only in his feet and was no longer in his hands. His pain was being managed by his primary care provider until his care could be transitioned to a pain clinic. Low-dose sirolimus was recommended, and the patient was agreeable to it.

Table 1 provides a timeline of the patient's major treatments.

## 3. Discussion

### 3.1. Rationale for Immunosuppression Therapy

The combination of anticoagulation (usually heparin), corticosteroids, and intravenous immunoglobulin and/or plasmapheresis is the most commonly used strategy in CAPS [13]. Immunosuppression has been a focus of treatment for CAPS, and the following rationales have been proposed for it. One strategy is direct antibody removal via plasmapheresis. Another is to decrease antibody production and normalize other B-cell disturbances. We will discuss each of these in more detail.

Thromboses and other clinical events occur as a result of antiphospholipid antibodies that interact with relevant targets, and beta 2 glycoprotein is the most relevant [13]. The structural change of beta 2 glycoprotein in response to inflammation or exposure to anionic phospholipids exposes the major B-cell epitope of this glycoprotein and allows binding to autoantibodies [13]. Thus, the direct removal of these offending antibodies via plasmapheresis could plausibly lead to a clinical benefit.

Apheresis is an extracorporeal medical procedure that involves removal of blood components temporarily utilizing centrifugal force, size, and structural differences of blood components and surface forces in microchannels as basis of separation [14]. A one-volume plasmapheresis involves the replacement of about 63% of the patient's plasma with a replacement fluid such as 5% albumin or, if indicated, plasma from blood donors [15]. Some centers extend it to a 1.5-volume exchange to replace about 78%, but the trade-offs include adding about an hour of nurse and machine time to an otherwise 2-hour procedure and increasing the citrate and replacement fluid volume by 50% while gaining only a 15% replacement [15].

According to the 2019 American Society for Apheresis guidelines, plasmapheresis is recommended as a category I indication for CAPS. However, this recommendation is based on limited evidence as noted in the introduction.

One source of evidence is the collection of case reports known as the CAPS Registry [16]. For example, using 242 CAPS episodes from that registry, one study reported recovery rates for CAPS using a variety of stand-alone and treatment combinations including anticoagulants, corticosteroids, plasma exchange, and intravenous immunoglobulins. Overall, recovery occurred in 56% of the episodes of CAPS, and death occurred in 44%. A higher recovery rate was found in the subset of cases where the patient was treated with plasma exchange plus anticoagulants plus corticosteroids (78%). According to that same study, patients treated with anticoagulants plus corticosteroids without plasma exchange had a recovery rate of 64% [17].

Plasma is often used as the replacement fluid in plasmapheresis when the patient is at risk for coagulopathic bleeding and/or thrombosis. The rationale for using plasma for CAPS in particular is to normalize the coagulation and complement systems as much as possible.

In addition to their role in antibody production, B cells alter T cell differentiation, regulate cytokines, and can contribute to fetal loss via decreased interleukin-3 production [13]. Blocking B cell-activating factor prevents disease onset and prolongs survival in mouse models [13]. Moreover, patients with antiphospholipid syndrome and venous thromboembolism have disturbed B-cell subset distribution compared to those with venous thromboembolism alone [13]. Thus, a therapy directed at mature B cells may yield a clinical benefit.

Rituximab is a chimeric human/murine monoclonal antibody that targets CD20 of memory and naïve B cells and results in depletion by antibody-dependent cytotoxicity, complement-mediated lysis, or apoptosis [18, 19]. It was originally made to treat mature B-cell malignancies, and many physicians have used it for conditions that are mediated by an offending autoantibody and alloantibody [19, 20]. Some indications for rituximab include rheumatoid arthritis, pemphigus, granulomatosis with polyangiitis, and microscopic polyangiitis [21]. In addition to case reports with various clinical responses, the available data on rituximab for antiphospholipid syndrome are limited to one uncontrolled prospective, open-label trial of 19 patients that did not show any significant therapeutic effect [22].

While some studies show no substantial change in autoantibody levels after rituximab, some of the features of CAPS may be due to high levels of acute phase reactants and cytokines [11]. Thus, by reducing the number of circulating B cells and normalizing their distribution, rituximab may lower the levels of these cytokines [11]. In other words, antibody titers alone may not tell the whole story.

Finally, other immunosuppressive agents that have been reported with variable clinical success include belimumab, bortezomib, eculizumab, and sirolimus [13]. For example, in patients with antiphospholipid syndrome (APS) nephropathy, there is evidence that sirolimus may result in improvement regarding kidney function and lesions of vasculopathy [12]. In the case of our patient, sirolimus was started several weeks after his initial presentation, and he benefited significantly. In retrospect, it may have been beneficial to start sirolimus earlier in the case of this patient.

### 3.2. Nuances and Limitations of Our Case Report

Our case report contains some nuances and limitations that should be addressed. First, the argatroban he received after extubation may have contributed to the duration of clinical benefit. However, the steroids and plasmapheresis most likely contributed the most to his rapid improvement given his dramatic change in clinical status in the days during and just after plasmapheresis. It is plausible that argatroban and rituximab provided him with a more long-term benefit.

In any event, this patient most likely cannot be compared directly to patients who received heparin, as this patient's history of heparin-induced thrombocytopenia precluded the use of heparin. Moreover, the history of heparin-induced thrombocytopenia and the inclusion of argatroban to the exclusion of heparin are not why this patient is noteworthy. The rare incidence of CAPS and the sparse evidence for plasmapheresis and especially rituximab for CAPS are the reasons why this patient is noteworthy. We address this use of argatroban in order to explain its rationale and to make sure that we do not mislead the reader into thinking that argatroban should be used instead of heparin in patients without this contraindication.

Second, we should emphasize that case reports, case series, and correlation studies are types of evidence known as event reporting or descriptive data, and controls are not available for comparison in these studies [23]. Thus, definite imputability is not possible to determine solely based on such event reporting data. However, such data are sometimes used by experts when writing guidelines and can also motivate interest in performing additional studies with more rigor [4].

Finally, some experts advocate for an update to the 2006 diagnostic criteria in order to include new clinical criteria and antibody specificities along with a scoring system that risk-stratifies patients [1, 24]. Despite these debates, this patient was diagnosed using the 2006 criteria.

## 4. Conclusion

We report a patient with catastrophic antiphospholipid syndrome who was successfully treated with corticosteroids, plasmapheresis, argatroban (instead of heparin due to a history of heparin-induced thrombocytopenia), and rituximab.

## Figures and Tables

**Table 1:** Timeline of major treatments.

Day	Event
−23	First presentation and admission for APS
−17	Rituximab dose #1 of 3
−16	Discharged
−3	Second presentation and admission for CAPS
0	TPE #1 of 4
1	TPE #2 of 4
2	TPE #3 of 4
5	TPE #4 of 4
6	Rituximab dose #2 of 3; discharged
13	Rituximab dose #3 of 3
19	Patient diagnosed with avascular necrosis (AVN)
21	Due for a fourth dose of rituximab; this dose was not given. The three prior doses amounted to a total of 900 mg of rituximab administered, and the patient's physicians decided that additional rituximab was unlikely to make a difference.
34	Sirolimus (2 mg daily) was started. Patient was on sirolimus for about 6 months.

## Data Availability

No data were used to support this study.

## Abbreviations

APS: Antiphospholipid syndrome
CAPS: Catastrophic antiphospholipid syndrome
TPE: Therapeutic plasma exchange.
